# The Prognostic Value of the Lymph Node in Oesophageal Adenocarcinoma; Incorporating Clinicopathological and Immunological Profiling

**DOI:** 10.3390/cancers13164005

**Published:** 2021-08-09

**Authors:** Noel E. Donlon, Maria Davern, Andrew Sheppard, Robert Power, Fiona O’Connell, Aisling B. Heeran, Ross King, Conall Hayes, Anshul Bhardwaj, James J. Phelan, Margaret R. Dunne, Narayanasamy Ravi, Claire L. Donohoe, Jacintha O’Sullivan, John V. Reynolds, Joanne Lysaght

**Affiliations:** 1 Department of Surgery, Trinity Translational Medicine Institute, Trinity College Dublin, St James’s Hospital, D08 W9RT Dublin, Ireland; davernma@tcd.ie (M.D.); SHEPPAA@tcd.ie (A.S.); POWERR8@tcd.ie (R.P.); oconnefi@tcd.ie (F.O.); heerana@tcd.ie (A.B.H.); ROKING@TCD.IE (R.K.); chayes5@tcd.ie (C.H.); Anshul.Bhardwaj@tcd.ie (A.B.); phelanj1@tcd.ie (J.J.P.); dunnem12@tcd.ie (M.R.D.); nravi@stjames.ie (N.R.); donohoe.claire@gmail.com (C.L.D.); osullij4@tcd.ie (J.O.); reynoldsjv@stjames.ie (J.V.R.); jlysaght@tcd.ie (J.L.); 2 Trinity St James’s Cancer Institute, St James’s Hospital, D08 W9RT Dublin, Ireland

**Keywords:** oesophageal adenocarcinoma, immune checkpoints, tumour-draining lymph node, prognostic, immunophenotyping, metastatic niche

## Abstract

**Simple Summary:**

Oesophageal cancer rates are increasing rapidly with patients often presenting at an advanced stage. The current approach to treatment involves radiotherapy, chemotherapy, or combination chemoradiotherapy with surgery; however, only a fraction of these patients will achieve meaningful responses. Therefore, there is a need to better understand the tumour and lymph node microenvironments to inform future treatment strategies. This study measured immune markers including immune checkpoint expression in tumour and lymph node tissue in oesophageal cancer patients and patient clinical outcomes, including survival time, response to treatment, and adverse events. We report herein that nodal metastases is of equal prognostic importance to clinical tumour stage and tumour regression grade in OAC and we observed a more immunosuppressive microenvironment in the tumour compared with the lymph node.

**Abstract:**

Response rates to the current gold standards of care for treating oesophageal adenocarcinoma (OAC) remain modest with 15–25% of patients achieving meaningful pathological responses, highlighting the need for novel therapeutic strategies. This study consists of immune, angiogenic, and inflammatory profiling of the tumour microenvironment (TME) and lymph node microenvironment (LNME) in OAC. The prognostic value of nodal involvement and clinicopathological features was compared using a retrospective cohort of OAC patients (*n* = 702). The expression of inhibitory immune checkpoints by T cells infiltrating tumour-draining lymph nodes (TDLNs) and tumour tissue post-chemo(radio)therapy at surgical resection was assessed by flow cytometry. Nodal metastases is of equal prognostic importance to clinical tumour stage and tumour regression grade (TRG) in OAC. The TME exhibited a greater immuno-suppressive phenotype than the LNME. Our data suggests that blockade of these checkpoints may have a therapeutic rationale for boosting response rates in OAC.

## 1. Introduction

Multimodal neoadjuvant therapy consisting of chemotherapy or chemoradiotherapy has become the standard-of-care for stage II–III cancer of the oesophagus and the oesophagogastric junction (OGJ). The potential benefits of neoadjuvant therapy include downstaging of the primary tumour, facilitating complete surgical resection and eradicating occult micrometastases [1]. However, neoadjuvant treatment results in a complete pathological response in just 15–30% of patients, and it is this subgroup that gains the survival benefit from systemic therapy [2,3]. It is therefore important to identify those that are at a high risk of recurrence after perioperative therapy as these patients may benefit from alternative regimens. As treatment is associated with substantial morbidity, early identification of non-responders could reduce the toxicity burden of minimally effective systemic therapies.

The lymphatic system has been recognised as a route of metastasis for over 150 years [4,5,6] and clinical nodal status (cN) is part of the preoperative staging of oesophageal cancer. Curative surgery includes lymph node dissection and [7] pathological staging of resected nodes (ypN) has been shown to independently predict survival for oesophageal cancer patients [8,9,10,11,12]. In addition to nodal status, histological tumour regression following treatment, measured by different tumour regression grade (TRG) scales, can predict overall survival [11,12,13,14,15]. However, in a secondary analysis of a randomised trial, only the nodal status was an independent prognostic factor [16]. The downgrading of nodal status following neoadjuvant treatment (where ypN is less than cN) also positively correlates with survival [17], particularly in patients that do not display a local response in their primary tumour [15]. However, it has been reported that those with nodal downstaging have shorter median survival than node-negative patients before treatment [17,18].

Along with their role in metastasis and as a prognostic factor, tumour-draining lymph nodes (TDLNs) are important in the anti-tumour immune response [19]. Priming of anti-tumour CD8^+^ T cells by dendritic cells (DCs) occurs in the lymph node, a critical step in the cancer immunity cycle that is potentiated by anti-CTLA-4 immune checkpoint blockade (ICB) [20,21]. However, TDLNs are rich in tumour-derived factors such as IL-6, TGF-β, prostaglandin E2, and VEGF, which promote an immunosuppressive milieu [19]. In melanoma, breast and cervical cancer, the lymph node microenvironment (LNME) promotes an immature and suppressive immune cell phenotype, through increased regulatory T cell (T_reg_) and myeloid-derived suppressor cell (MDSC) infiltration as well as by a generalized state of enhanced T cell anergy. In addition, lymph node (LN)-resident DC subsets show lower levels of activation compared with that of migratory DC subsets [22,23,24], subsequently attenuating T cell activation, thus allowing for subsequent tumour progression and metastasis [25]. In this altered cytokine milieu, CD4^+^ T cells do not differentiate into effector T cells but instead differentiate into peripheral T_reg_ cells that restrain anti-tumour immunity [26]. The LNME is emerging as an important substrate for immune checkpoint blockade (ICB); in mouse models, ablation or surgical resection of sentinel lymph nodes reduces immune cell infiltration in the primary tumour and reduced the efficacy of anti-PD-1 and anti-PD-L1 therapy [27]. These studies highlight the complex yet critical role of TDLNs in promoting or inhibiting anti-tumour immunity and mediating response to ICB [28]. Furthermore, elective nodal irradiation in mice reduces chemokine expression in the tumour microenvironment (TME) and subsequent intratumoural CD8^+^ T cell infiltration resulting in reduced ICB efficacy [29]. This also has therapeutic implications, as local delivery of ICB to TDLNs in mice had similar efficacy to intratumoral delivery [30]. However, the LNME has yet to be examined in the context of oesophagogastric adenocarcinoma. An understanding of the LNME niche would help inform the development of rational immunotherapeutic strategies to boost response rates to ICB and conventional therapies in oesophagogastric adenocarcinomas [31,32,33].

In this study, we investigated the prognostic implications of clinical and pathologic nodal status in a large single-centre cohort of patients with locally advanced resectable oesophagogastric cancer. Additionally, we profiled immune checkpoint (IC) expression on T cells residing in the TDLN and infiltrating-tumour tissue of oesophagogastric adenocarcinoma patients. Furthermore, the inflammatory, angiogenic, cytokine, and chemokine profile of the LNME and TME in oesophagogastric cancer patients is investigated and correlated with clinicopathologic outcomes. This information will help provide a fundamental understanding of the LNME and TME in oesophagogastric adenocarcinoma patients in order to better inform future therapeutic approaches.

## 2. Methods

### 2.1. Ethics Statement

Ethical approval was granted from Tallaght/St James’s Hospital Ethics Committee. Informed written consent was obtained for all the sample and data collection, which was carried out using best clinical practice guidelines. All procedures followed were in accordance with the ethical standards of the responsible committee on human experimentation (institutional and national) and with the Helsinki Declaration of 1975, as revised in 2008. Patient samples were pseudonymised to protect privacy rights.

### 2.2. Specimen Collection

All patients who consented to fresh specimen collection were enrolled between 2018 and 2020. Tumour biopsies (*n* = 9) along with matched TDLN tissue biopsies (*n* = 6) were obtained from oesophageal adenocarcinoma patients at time of surgical resection at St James’s Hospital, Dublin, Ireland. Paraoesophageal lymph nodes were confirmed to be negative for metastatic disease by formal laboratory histological analysis.

### 2.3. LN and Tumour Tissue Digestion

Biopsies were enzymatically digested to perform single-cell phenotyping. Briefly, tissue was minced using a scalpel and digested in collagenase solution (2 mg/mL of collagenase type IV (Sigma, MO, USA) in Hanks Balanced Saline Solution (GE healthcare, Wauwatosa, WI, USA) supplemented with 4% (*v*/*v*) foetal bovine serum) at 37 °C and agitated at 1500 rpm on an orbital shaker. Tissue was then filtered using 70 uM nylon mash filter and washed with FACs buffer (PBS containing 1% foetal bovine serum and 0.01% sodium azide). The resulting single cell suspensions were then stained for flow cytometry.

### 2.4. Flow Cytometry Staining

LN and tumour tissue biopsies were stained with zombie aqua viability dye (Biolegend, San Diego, CA, USA) and the following cell surface antibodies: PD-L1 PE and CD8 BV421 (BD Biosciences, Franklin Lakes, NJ, USA), TIM-3 Viobright FITC and CD3 APC (Miltenyi, Bergisch Gladbach, Germany), TIGIT PE/Cy7 and PD-1 APC/Cy7 (Biolegend, San Diego, CA, USA) and CD4 PerCpCy5.5 (eBiosciences, San Diego, CA, USA). Cells were fixed with 1% paraformaldehyde solution (Santa Cruz, TX, USA), washed and resuspended in FACs buffer, and acquired using BD FACs CANTO II (BD Biosciences) using Diva software vX and analysed using FlowJo v10 software (TreeStar Inc., Ashland, OR, USA).

### 2.5. Generation of Lymph Node Conditioned Media (LNCM) and Tumour Conditioned Media(TCM)

LN (*n* = 6) and tumour tissue (*n* = 6) biopsies were cultured in a 12-well plate in 1 mL of L-15 (Leibovitz) Lonza™ BioWhittaker™ (Basel, Switzerland) X-vivo media for 24 h ex vivo at 37 °C, 5% CO_2_. LN tissue was divided into four equal quadrants to ensure adequate distribution of immune cells. LN were confirmed to be benign through formal histological assessment by the St James’ Hospital Histopathology department. The resulting lymph-node-conditioned media (LNCM) and tumour-conditioned media (TCM) was harvested and stored at −80 ^°^C until required for further experimentation.

### 2.6. Quantification of Serum Immune Proteins

A panel of 54 angiogenic, vascular injury, pro-inflammatory, cytokine, and chemokine mediators were quantified by 54-plex ELISA in LNCM and TCM according to the manufacturer’s instructions (Meso Scale Diagnostics, Rockville, MA, USA). The following secretions were quantified in the LNCM and TCM: CRP, Eotaxin, Eotaxin-3, bFGF, Flt-1, GM-CSF, ICAM-1, IFN-γ, IL-10, IL-12/IL-23p40, IL-12p70, IL-13, IL-15, IL-16, IL-17A, IL-17A/F, IL-17B, IL-17C, IL-17D, IL-1RA, IL-1α, IL-1β, IL-2, IL-21, IL-22, IL-23, IL-27, IL-3, IL-31, IL-4, IL-5, IL-6, IL-7, IL-8, IL-8 (High Abundance), IL-9, IP-10, MCP-1, MCP-4, MDC, MIP-1α, MIP-1β, MIP-3α, PlGF, SAA, TARC, Tie-2, TNF-α, TNF-β, TSLP, VCAM-1, VEGF-A, VEGF-C and VEGF-D. All assays were run as per the manufacturer’s instructions, with an overnight supernatant incubation protocol used for all assays except angiogenesis panel 1 and vascular injury panel 2, which were run according to the same day protocol. TCM and LNCM were run undiluted on all assays except vascular injury panel 2, where a one in four dilution was used, as per previous optimisation experiments. Secretion data for all factors was normalised to cell lysate protein content determined using a BCA assay (Pierce, Waltham, MA, USA).

### 2.7. Statistical Analysis

Statistical analysis was performed using SPSS^®^ (version 18.0) software (SPSS, Chicago, IL, USA) and R 2.13.2. A significance level of *p* < 0.05 was used for all analyses and all *p* values reported are two-tailed. The Kaplan–Meier method and the log rank test was used to assess differences in survival between groups. Survival time was measured from the date of first treatment to the date of death or last follow-up. Independent variables were entered into a multivariable Cox proportional hazards model, variables found at univariable analysis to have a *p* value < 0.05 were entered into the multivariable model. Continuous variables were compared using unpaired *t*-tests. Association of categorical variables was assessed using χ^2^ test.

Wilcoxon rank test was utilized to compare checkpoint, cytokine, and markers of angiogenesis expression between the lymph node and the tumour compartment.

## 3. Results

### 3.1. Nodal Status and Pathological Complete Response Are Equally Important Prognostication Markers in OAC in Predicting Overall Survival Time

Data for 702 patients were obtained from a prospectively maintained database between 2001 and 2016, of these 356 were classified as node negative, with a higher proportion of the N0 cohort having a Siewert type I tumour (60%). An en bloc radical oesophagectomy was performed in 50% of N0, 66% N1, 48% of N2, and 53% of N3 (Table 1). On pathologic assessment of resected specimens, N0 had earlier T stage disease, with 54% being (y)p or pT0/T1, compared with 18% for N1, 9% for N2, and 7% for N3 (*p* < 0.001). N0 and N1 were significantly associated with Barrett’s oesophagus, 55% and 49%, respectively (*p* < 0.001), with signet ring and mucinous features more frequent in N2 and N3. N0 was associated with less adverse features of tumour when compared to those with nodal positivity. Adverse features consisted of poor differentiation, perineural, lymphatic, and vascular invasion, with a novel three-grouping stratification of 0, 1–2, and 3–4 adverse features previously described by our department [34]. Linking adverse pathology with nodal status, 52%, 42%, and 6% of N0 were in the 0, 1–2, and 3–4 group, respectively, compared with 16%, 58%, and 26% for N1; 6%, 51%, and 43% for N2; and 5%, 44%, and 51% for N3 (*p* < 0.001). Chemoradiation was the most commonly utilised neoadjuvant treatment modality across all nodal status. Mandard TRG after neoadjuvant therapy was significantly better in N0, with 25% either TRG 1 or 2, compared with 14% in N1, 12% in N2, and 8% in N3. The clinical and pathologic demographics per nodal burden are outlined in Table 1.

Adverse features of tumour biology including perineural invasion, vascular invasion, differentiation nodal positivity, and neoadjuvant treatment were significant by multivariate analysis on overall survival. (Table 2).

**Figure 1:**
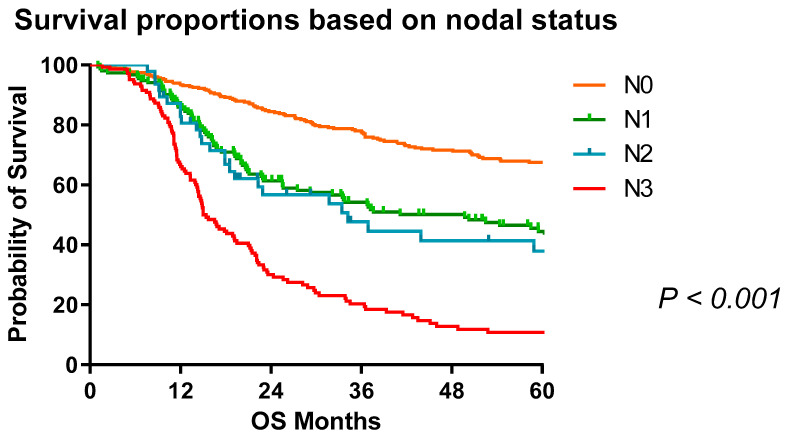
Survival proportions of OAC patients based on nodal status (N0 = node negative, N1 = 1–2 Nodes, N2 = 3–6 nodes, N3 ≥ 7). Any nodal disease burden significantly reduces overall survival with proportional reduction per stage of dissemination. Median survival: N0, not reached; N1, 49.7 months; N2, 34.23 months; N3, 15.43 months.

For the entire cohort classified by nodal status (Figure 1), the median overall survival was not reached for N0, 49.7 months for N1, 34.2 months for N2, and 15.4 months for N3 with 5-year survival of 67%, 45%, 38%, and 11%, respectively (*p* < 0.001). The median overall survival was 58.27 months (CI 46.28–70.26). The five year survival for node negative disease in 67%, 45% for N1 disease, 38% for N2, and 11% for N3 disease (Table 3).

**Figure 2:**
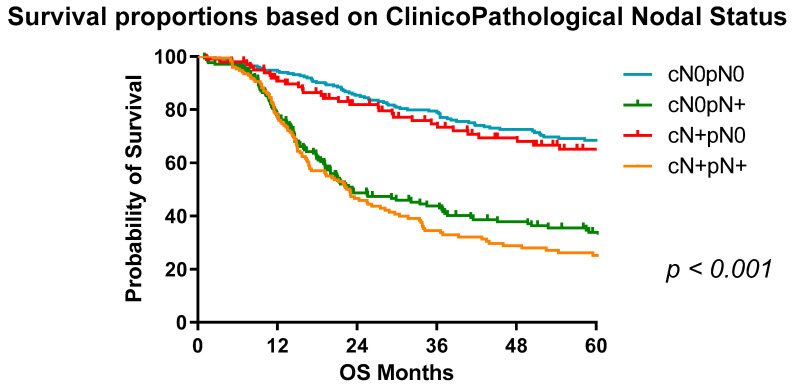
Survival proportions of OAC patients based on clinicopathological nodal status. Median survival: cN0pN0, not reached; cN+pN0, 124.6 months; cN0pN+, 22.93; cN+pN+, 22.5 months.

Patients with positive clinical nodal status (based on endoscopic ultrasound and/or positron emission tomography (PET)) but pathologically node-negative had improved survival compared to those who were clinically and pathologically node-positive (*p* < 0.001) (Figure 2). Important caveats to this finding include the fact that clinical staging is carried out at time of diagnosis and pathological staging is carried out post-surgical resection. There are a multitude of reasons for such discrepancies such as an increase in tumour burden as a consequence of adverse biology or treatment refractory disease reducing responses to neoadjuvant therapy. Similarly, clinical staging through endoscopy, conventional CT TAP and PET/CT imaging cannot detect microscopic disease; therefore, final pathological grading is definitive.

The five-year survival for clinically node negative, pathologically node negative disease is 68%, compared to 61% in those with clinically node positive, pathologically node negative disease. In those with clinically and pathologically node positive disease, the five-year survival is 26% (Table 4).

**Figure 3:**
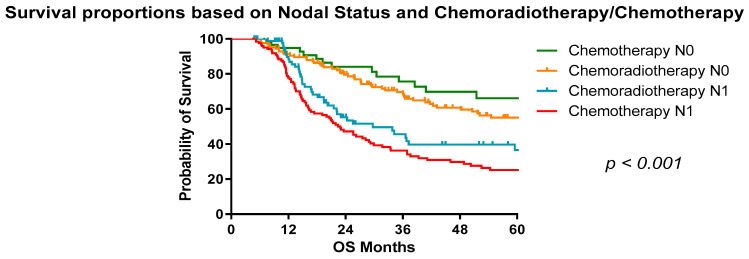
Survival proportions of OAC patients based on nodal status and treatment received (chemoradiotherapy/chemotherapy). Median survival: pN0 chemotherapy, 79.2 months; pN0 chemoradiotherapy, 71.64 months; pN+ chemotherapy, 22.9 months; pN+ chemoradiotherapy, 29.73 months.

**Figure 4:**
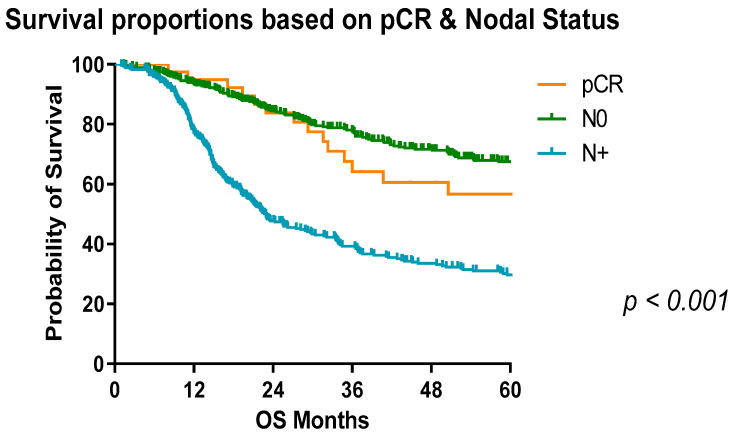
Survival proportions of OAC patients based on nodal status and pathologic complete response. pCR refers to complete pathological response on histological analysis, N0—node negative, N+—node positive. Significantly reduced overall survival with nodal disease. Median survival: pCR, not reached; N0, 124.6 months; N+, 22.9 months.

In patients treated with neoadjuvant chemotherapy, the median survival in patients who were pN0 was 79.2 months compared with 71.64 months in those treated with chemoradiotherapy. On the contrary, in patients who were pN+ treated with neoadjuvant chemotherapy, the median survival was 22.9 months compared to 29.73 months in those treated with chemoradiotherapy (Figure 3). Interestingly, patients who were node negative had a higher median survival than those with a pCR on final histological analysis to neoadjuvant therapy and significantly better than those who had nodal positivity (*p* < 0.001) (Figure 4). The 5-year overall survival for those with node positive disease and treated with chemotherapy was 37% compared with 25% with chemoradiotherapy (Table 5).

### 3.2. Inhibitory Immune Checkpoint Receptors and Ligands Are Expressed at Significantly Higher Levels on Tumour-Infiltrating T Cells Compared to Tumour-Draining Lymph Nodes

Inhibitory ICs play key roles in restraining anti-tumour immunity and with the recent approval of two anti-PD-1 inhibitors, pembrolizumab and nivolumab, for treating oesophagogastric adenocarcinomas, it is important we develop a deeper understanding of IC expression profiles in adenocarcinoma patients. We profiled IC expression on T cells infiltrating OAC tumour tissue and in the TDLN to determine the level of target expression in multimodal therapy.

**Figure 5:**
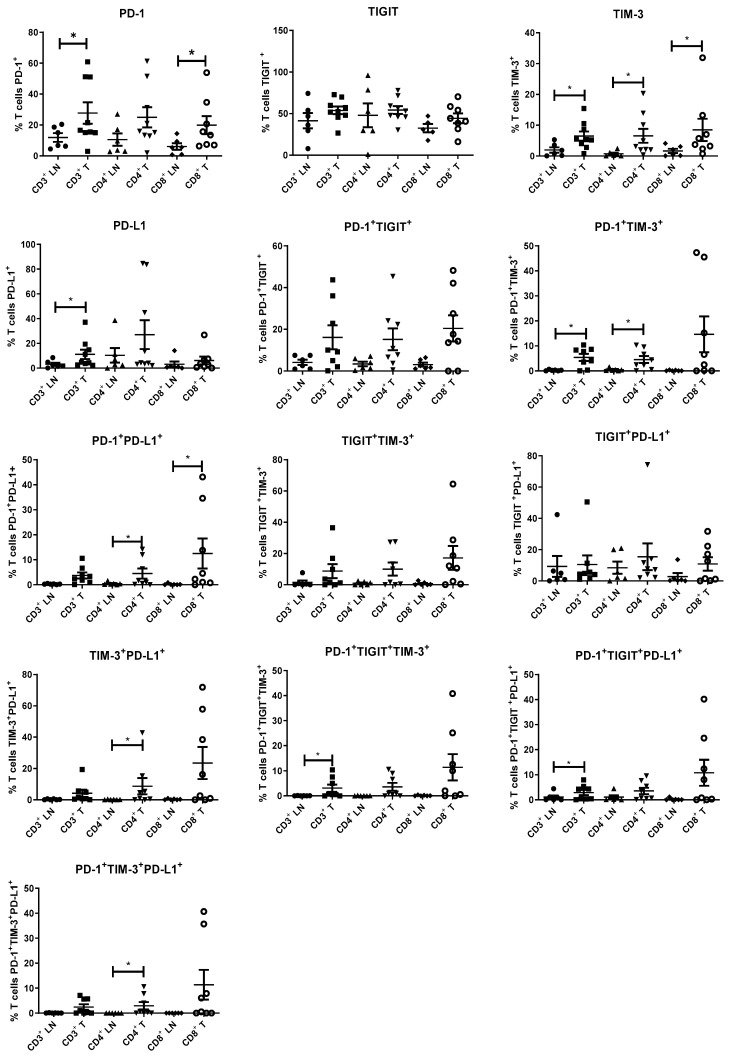
Inhibitory IC receptors and ligands are expressed at significantly higher levels on tumour-infiltrating T cells compared to T cells present in tumour-draining lymph nodes. CD3^+^, CD3^+^CD4^+^, CD3^+^CD8^+^ cells in tumour-draining lymph nodes (*n* = 6) and infiltrating tumour tissues (*n* = 6) in OAC patients were screened for the surface expression of PD-1^+^, TIGIT^+^, TIM-3^+^ and PD-L1^+^ ex vivo by flow cytometric analysis. Tumour draining lymph nodes and tumour specimens were post-treatment and taken at surgical resection. Wilcoxon rank test was used to compare expression between lymph node and tumour compartment; * *p* < 0.05. LN: lymph node and T: tumour.

Overall, a higher percentage of T cells expressing inhibitory ICs was found within tumour tissues compared to TDLNs and this pattern was consistent across CD4^+^ T helper and CD8^+^ cytotoxic T cell compartments (Figure 5). A significantly higher percentage of CD3^+^CD8^+^PD-1^+^ cells were found in tumour tissue compared with TDLNs (median 18.2 (range 7.41–46.49) vs. 5.52 (range 0.75–13.65), *p* < 0.05). There was also a significantly higher percentage of CD3^+^TIM-3^+^ (median 7.1 (range 5.23–15.4) vs. 1.33 (range 0.06–5.28)), CD3^+^CD4^+^TIM-3^+^ (median 7.51 (range 0.35–20.3) vs. 0.36 (range 0.05–2.41)) and CD3^+^CD8^+^TIM-3^+^ (median 6.37 (range 2.31–31.9) vs. 1.3 (range 0.06–4.07)) intratumourally compared to the lymph node (*p* < 0.05). There was a significantly higher expression of CD3^+^ PD-L1^+^ cells (median 12.95 (range 2.19–37)) in the tumour compared to (median 2.07 (range 0.15–8.46)) in the lymph node (*p* < 0.05). The expression of CD3^+^ PD-1^+^TIM-3^+^ (median 5.91 (range 0.01–10.39) vs. 0.27 (range 0–0.63), *p* < 0.05), and CD3^+^CD4^+^ PD-1^+^TIM-3^+^ (median 3.41 (range 0–10.3) vs. 0.33 (range 0–1.6), *p* < 0.05), was significantly higher in the tumour than in the lymph node (Figure 5). There was a significantly higher expression of CD3^+^ CD4^+^PD-1^+^PD-L1^+^ cells intratumourally (median 4.35 (range 0.2–14.18) compared to the lymph node (median 0.33 (range 0–1.6) *p* < 0.05). Similarly, CD3^+^CD8^+^ PD-1^+^PD-L1^+^ cells were also found at a significantly higher frequency in tumour-infiltrating tissue compared with TDLNs (median 1.62 (range 0.02–34.6) vs. 0.12 (range 0–0.87) *p* < 0.05), similarly for CD3^+^CD4^+^TIM-3^+^PD-L1^+^ cells (median 5.03 (range 0.02–42.8) vs. 0.11 (range 0–0.41), *p* < 0.05) (Figure 5). There was also a significantly higher expression intratumourally of CD3^+^ PD-1^+^TIGIT^+^TIM-3^+^ (median 5.63 (0.02–7.58) vs. 0.03 (0–0.09) as well as CD3^+^ PD-1^+^TIGIT^+^ PD-L1^+^ (median 6.02 (range 0.42–7.99) vs. 1.93 (range 0.01–4.44) (*p* < 0.05). Significantly higher frequencies of CD3^+^CD4^+^PD-1^+^TIM-3^+^PD-L1^+^ cells intratumorally (median 0.68 (range 0.01–10.6) vs. 0.03 (range 0–5.65), *p* < 0.05), (Figure 5).

The levels of anti-tumour cytokines IFN-γ were lower in the TME (median 13.63 pg/mL (range 1.15–43.83) compared to the LNME (median 41.27 pg/mL (range 18.1–182.2) but did not achieve significance by multivariate analysis (Figure S1). However, the levels of IL-9 and IL-27 cytokines were higher within the TME (median 6.55 pg/mL (range 2.14–9.86) and 25.92 pg/mL (range 17.18–337.2)) compared to the LNME (1.31 pg/mL (range 0.41–4.82) and 12.88 pg/mL (range 0.77–257), respectively, of OAC patients, but again did not achieve significance by multivariate analysis (Figure S1).

The LNME and TME was assessed for pro-angiogenic and vascular damage analytes and the levels of soluble bFGF were higher within the TME 124.8 pg/mL (range 66.06–610.8) compared with the LNME (median 34.97 pg/mL (range 22.35–72.94) but were not significant. Interestingly, PIGF was higher within the LNME 116.3 pg/mL (range 18.78–162.6) than the TME 10.15 pg/mL (range 4.61–82.11) of OAC patients but was not significant. The levels of all other analytes were detected at comparable levels between the LNCM and TCM from OAC patients, and these cytokines, chemokines, and markers of vascular injury and angiogenesis are available as Table S1.

## 4. Discussion

While there are limitations to the current study, primarily the cohort size of fresh patient samples, this should be borne in mind when considering the conclusions of the study, as this limits the ability to perform clinical correlative work. We have demonstrated in a very large cohort of OAC patients (*n* = 702) that the presence of nodal metastases has equivalent prognostic value to that of clinical tumour stage and TRG in OAC patients. This further highlights that the TDLN may play a pivotal role in orchestrating anti-tumour responses and subsequent treatment response in OAC patients and complements previous studies.

Identifying patients at risk of relapse following surgery for OAC remains a challenge, but histological assessment of surgical specimens is an attractive means of predicting response. TRG 1–2, as determined by the Mandard or Becker systems, has a robust association with overall survival (OS) and disease-free survival in resected specimens [11,12,13,14,15]. The pathological tumour extent in the resected specimen (pT) is also associated with recurrence and survival in OAC. Our results are in agreement with this, as TRG and pT stage are significant prognostic markers in patients following neoadjuvant treatment. In a post hoc analysis of the MAGIC trial of perioperative chemotherapy in resectable oesophagogastric cancer, both TRG1–2 and nodal status were negatively related to survival [16]. However, after multivariate adjustment, it was only the presence of lymph node metastasis that was an independent predictor of OS (HR: 3.36; 95% CI, 1.70 to 6.63). Interestingly, a machine learning model developed to predict the risk of recurrence following neoadjuvant treatment found that the number of lymph node metastases was the most important variable in the model, with lymphovascular invasion second [35]. This is similar to our findings as we demonstrate that nodal status has equivalent prognostic value compared to pT and TRG in patients treated with both chemoradiotherapy and perioperative chemotherapy.

A greater understanding of the relationship between the immune microenvironments of the tumour and lymph node will be useful in understanding the response to chemoradiotherapy and future immunotherapy. Harnessing the power of TLDNs may also boost the clinical outcomes following ICB. Indeed, ongoing clinical trials are investigating the role of ICB alongside neoadjuvant chemoradiotherapy in locally advanced, operable gastroesophageal adenocarcinoma (NCT02730546, NCT03044613) [31]. The LNME is suggested to be an immunosuppressive niche. One study using a mouse model of lung adenocarcinoma demonstrated that the LNME skews tumour antigen-specific CD4^+^ differentiation into regulatory T cells, thus promoting tumour immune escape [26]. In human melanoma patients, lymph node metastases are associated with the suppression of the CD1a^+^ DC subset, a cellular population that is efficient at cross-presenting neoantigens to CD8^+^ T cells. This immunosuppressive feature of the LNME was correlated to local tumour recurrence [5]. This also corresponded to an increased CD4:CD8 T cell ratio, and enrichment of regulatory T cells in the local nodal microenvironment in metastatic disease. This finding has since been replicated in breast and cervical cancer [24,36], where DC suppression was associated with impaired T cell effector function.

Our data is broadly in agreement with these studies; however, we not only analysed the immune profile of the LNME, but also compared it to the immune microenvironment of primary tumour samples, demonstrating that the TME exhibited greater immunosuppression compared to the LNME. We found that the TME had higher levels of infiltrating T cells co-expressing multiple ICs, which typically denote T cell exhaustion and functional impairment, compared to the LNME. The presence of IC expression by T cells in the LNME was previously reported in one study of cervical cancer patients, but it was not compared with the TME [24]. Using cytokine profiling, we also found that the TME had lower levels of IFN-γ, compared to the LNME. This suggests a more suppressive immune landscape, as IFN-γ promotes DC antigen presentation as well as encouraging CD8 T cell responses [37]. Interestingly, in the KEYNOTE-028 clinical trial, an IFN-γ gene expression signature was predictive of response to ICB in OC patients, underscoring its role in the immune response to OAC [38]. Levels of IL-9 and IL-27 were higher in the LNME compared to the TME, with both having mixed or tumour-promoting effects in terms of cancer progression [39]. Collectively, our findings indicate greater immunosuppression in the TME compared to the LNME, which could explain why intact TDLNs are indispensable for the clinical activity of ICB in mouse models [27,28]. However, the presence of exhausted T cells within a tumour does point toward a pre-existing antigen-specific anti-tumour immune response, a pre-requisite for response to ICBs.

The levels of immunosuppression in the LNME may also have clinical implications, as the presence of suppressed DC subsets in the TDLNs are negatively associated with recurrence and disease-free survival in melanoma and breast cancer [22,25]. Furthermore, PD-1, TIM-3, and TIGIT expression may represent mechanisms of immune escape in OAC and perhaps ICBs targeting PD-1, TIM-3, and TIGIT may represent a more effective and personalised rational approach for treating oesophagogastric cancer patients.

Although cancer cells were traditionally thought to spread to distant organs from lymph nodes through lymphatic vessels, recent preclinical studies suggest that cancer cells can invade local nodal blood vessels to enable a mixed haematogenous metastatic dissemination [4,5]. Thus, markers of angiogenesis and blood vessel formation may prove useful in understanding the metastatic potential of the TDLN. Levels of pro-angiogenic and wound healing markers including bFGF and PIGF were higher within the LNME than the TME. These mediators play important roles in promoting wound healing, angiogenesis, and tumour growth and therefore may play a role in remodelling the TDLN into a tumour permissive niche enabling nodal metastasis. The levels of the vascular damage protein bFGF were higher within the TME compared with the LNME. Known functions of bFGF include the enhancement of tumour cell proliferation, survival, motility, and wound healing [40]. Previous studies in oesophageal cancer demonstrated that bFGF overexpression is associated with a risk of tumour recurrence and reduced OS post-surgical resection, suggesting bFGF may play an important role in TME remodelling and enhancement of tumour progression [41,42].

Collectively, these data highlight a potentially detrimental role of tumour-promoting inflammation in OAC pathogenesis. Tumour-promoting inflammation is one of the enabling hallmarks of cancer and plays important roles in therapy resistance and tumour progression [43]. Interestingly, our study suggests that inflammation within the LNME may be beneficial as the levels of pro-inflammatory cytokines and chemokines are negatively correlated with advanced tumour stage and further supports the hypothesis of tumour-promoting inflammation. The TDLN is a key orchestrator of anti-tumour immunity; therefore, a heightened state of immune activity would be expected within the LNME. This also highlights that anti-cancer immune-promoting therapies should be developed to target the TDLN and not just the TME.

The role of Th17 cells in cancer is controversial, with studies suggesting both pro- and anti-tumour effects in diverse cancer types [44]. There is an abundance of work that detailed enrichment of Th17 cells in the TME of both gastric and oesophageal cancer [45,46], which still requires additional research to delineate the exact role of these cells in the TME and TDLN.

There are a number of limitations in this study in addition to the small cohort size for immunophenotyping, including the fact that fresh lymph node and tumour tissue samples were obtained at surgical resection, which is post-multimodal treatment. The impact of treatment can alter the microenvironment of the tumour and the lymph nodes within in the treatment field. However, understanding how treatment can affect the TME and LNME will have a significant bearing on the development of appropriate immunotherapeutic targeting. Whilst we have shown significant immunological differences between the tumour and the draining lymph node in this small cohort, additional larger studies using treatment naïve samples will be required to determine the real clinical significance of these potential therapeutic targets.

## 5. Conclusions

In summary, nodal involvement, and clinical and pathological tumour staging are significant prognostic indicators for OAC. These results also highlight the immunosuppressive nature of the TME demonstrated by higher percentages of T cells co-expressing multiple ICs in the TME compared to the LNME. The data are timely with the recently published Checkmate 577 trial advocating the use of immunotherapy in the adjuvant setting in carcinoma of the oeosphagogastric junction. Our data supports the hypothesis that combination therapeutic blockade of these aforementioned checkpoints may have a therapeutic rationale for boosting response rates in OAC.

## Figures and Tables

**Table 1:** Clinical characteristics for patient cohort included in study.

Clinical Characteristics	pN0	pN1	pN2	pN3	*p*-Value
	*N* = 356	*N* = 154	*N* = 47	*N* = 145	
Age, mean (SD)	63.62 (10.47)	62.97 (10.03)	65.02 (10.09)	63.22 (10.85)	0.64
Sex, (*n*)					
Female	62	24	6	28	
Male	294	130	41	117	0.71
BMI, kg/m^−2^, mean (SD)	27.47 (4.58)	27.32 (4.35)	28.65 (4.62)	26.81 (4.54)	0.78
Obese	218	97	33	74	0.15
Current smoker	156	6	18	49	0.38
Diabetes	4	18	8	16	0.74
Hypertension	106	40	9	39	0.43
Dyslipidemia	62	20	9	12	0.046
ASA grade,					
ASA I	139	57	13	51	
ASA II	166	78	23	77	
ASA III	51	19	11	17	0.37
Siewert Classification					
Siewert I	217	76	19	48	
Siewert II	77	48	11	39	
Siewert III	62	30	17	58	<0.001
Treatment characteristics					
Treatment pathway:					
Neoadjuvant therapy	186	106	24	79	
Surgery first	170	48	23	66	0.005
Neoadjuvant regimen:					
Chemotherapy	59	39	7	39	
Chemoradiation	127	67	17	40	0.04
Operation type					
2-stage esophagectomy	197	101	23	77	
3-stage esophagectomy	27	10	0	15	
Transhiatal esophagectomy	78	12	7	7	<0.001
Extended total gastrectomy	54	31	17	46	
Clinical and Pathologic					
Clinical T stage					
1	112	14	2	4	
2	58	21	6	19	
3	183	114	38	114	
4	3	5	1	8	<0.001
Clinical N stage					
0	256	87	28	57	
1	87	57	15	73	
2	12	9	3	11	
3	1	1	1	4	<0.001
Proximal Margin Clear	353	149	46	133	<0.001
Distal Margin Clear	353	146	44	131	<0.001
Radial Margin Clear	305	112	36	71	<0.001
Lymphatic invasion	51	100	35	119	<0.001
Venous invasion	65	66	27	92	<0.001
Perineural invasion	25	35	16	50	<0.001
Poor or undifferentiated grade, (poor versus other)	110	59	23	84	<0.001
Barrett’s oesophagus	173	58	17	34	<0.001
Signet Ring	25	13	4	32	<0.001
Mucinous features	33	18	6	34	<0.001
Barrett’s in Tumour	194	75	18	47	<0.001
Adverse feature grading:					
No adverse features	184	25	3	7	<0.001
1 or 2 adverse features	151	90	24	64	
3 or 4 adverse features	21	39	20	74	
Pathologic stage:					
(y)pT0	37	1	1	3	
(y)pT1	154	26	3	7	
(y)pT2	67	29	7	14	<0.001
(y)pT3	92	87	34	105	
(y)pT4	6	11	2	16	
Tumour regression grade N (%)					
TRG 1	40	1	1	1	
TRG 2	51	20	5	12	
TRG 3	54	40	7	19	
TRG 4	33	33	8	30	
TRG 5	9	13	4	17	<0.001
CAP					
R0	335	133	37	95	
R1	21	21	10	50	<0.001
RCPATH					
R0	302	109	34	79	
R1	54	45	13	66	<0.001
Recurrence	97	83	29	115	<0.001
Median Survival (Months)	Not reached	49.7	34.23	15.43	<0.001
1 Year Survival	94	86	83	67	
3 Year Survival	78	54	48	21	<0.001
5 Year Survival	67	45	38	11	

**Table 2:** Univariable and multivariable analysis for overall survival.

Variable	Univariable			Multivariable		
	HR	CI	*p* Value	HR	CI	*p* Value
Barretts history	1.44	1.138–1.821	<0.001	1.680	0.919–3.072	0.09
Neoadjuvant treatment	1.528	1.211–1.928	<0.001	1.434	1.177–1.746	<0.001
pN (node negative vs. positive)	1.577	1.104–1.747	<0.001	1.399	1.151–1.703	<0.001
Perineural invasion	1.601	1.257–2.039	<0.001	1.475	1.125–1.935	0.005
Vascular invasion	1.548	1.219–1.465	<0.001	0.527	0.316–0.879	0.01
Differentiation (poor versus other)	1.584	1.245–2.017	<0.001	1.582	1.098–1.595	0.02

**Table 3:** Overall number of surviving OAC patients based on nodal status (pathological post-resection).

Survival	N0			N1			N2			N3		
(Years)	No at Risk	Deaths	% Survival	No at Risk	Deaths	% Survival	No at Risk	Deaths	% Survival	No at Risk	Deaths	% Survival
1	356	22	94	154	21	86	47	8	83	145	46	67
3	317	49	78	124	43	54	38	14	48	89	58	21
5	225	28	67	67	11	45	15	3	38	22	10	11

**Table 4:** Overall survival of OAC patients based on clinicopathological nodal status.

Survival	cN0pN0			cN+pN0			cN0pN+			cN+pN+		
(Years)	No at Risk	Deaths	% Survival	No at Risk	Deaths	% Survival	No at Risk	Deaths	% Survival	No at Risk	Deaths	% Survival
1	256	13	95	100	9	91	172	38	77	174	37	78
3	231	35	79	86	14	75	126	52	44	125	63	35
5	166	21	68	59	7	65	61	13	34	43	11	26

**Table 5:** Overall survival of OAC patients based on nodal status and treatment received (chemoradiotherapy/chemotherapy).

Survival	Chemotherapy N0			Chemoradiotherapy N0			Chemotherapy N+			Chemoradiotherapy N+		
(Years)	No at Risk	Deaths	% Survival	No at Risk	Deaths	% Survival	No at Risk	Deaths	% Survival	No at Risk	Deaths	% Survival
1	59	3	95	127	11	91	85	8	90	124	26	78
3	50	8	76	112	25	70	66	28	46	89	45	36
5	27	3	66	73	14	55	23	4	37	34	10	25

## Data Availability

The data presented in this study are available in the article and Supplementary Material.

## Supplementary Materials

The following are available online at https://www.mdpi.com/article/10.3390/cancers13164005/s1, Figure S1: Chemokines, cytokines, Markers of angiogenesis and vascular injury. Table S1: Analyte in matched tumour and lymph node.
