# Supplementary material for: The Prognostic Value of the Lymph Node in Oesophageal Adenocarcinoma; Incorporating Clinicopathological and Immunological Profiling
Source: Cancers (Basel). 2021 Aug 9;13(16):4005. doi: 10.3390/cancers13164005 (PMC8391676; doi:10.3390/cancers13164005)
Supplement: Supplementary file 1 [file cancers-13-04005-s001.zip › cancers-1251851-supplementary.pdf]

Supplementary Materials: The Prognostic Value of the Lymph Node in Oesophageal Adenocarcinoma; Incorporating Clinicopathological and Immunological Profiling

Noel E Donlon, Maria Davern, Andrew Sheppard, Robert Power, Fiona O Connell, Aisling Heeran, Ross King, Conall Hayes, Anshul Bhardwaj, James J Phelan, Margaret R Dunne, Narayanasamy Ravi, Claire L Donohoe, Jacintha O' Sullivan, John V Reynolds and Joanne Lysaght

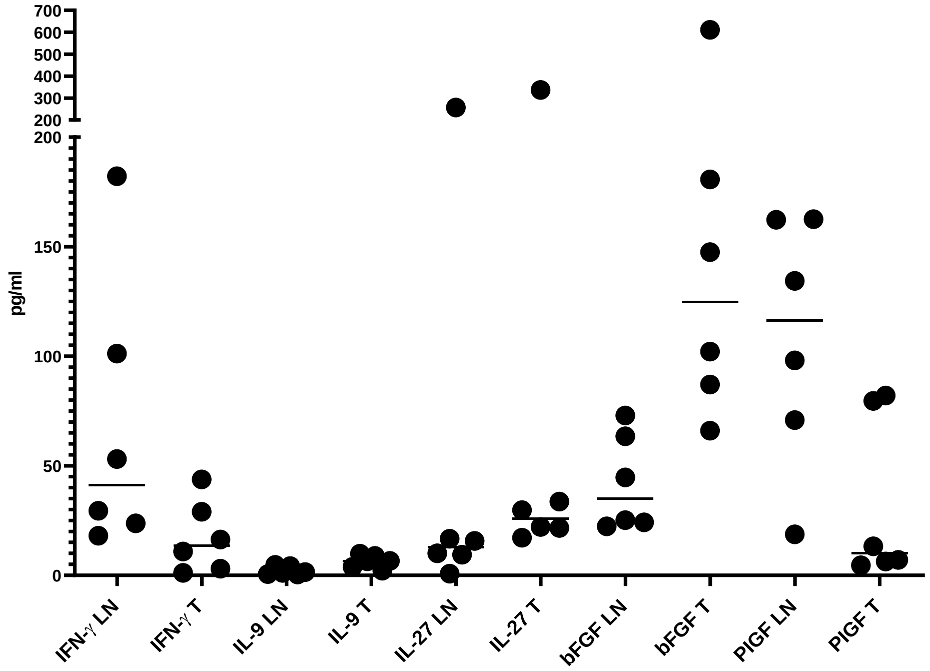

Figure S1. Chemokines, cytokines, Markers of angiogenesis and vascular injury; The levels of IFN-Y, IL-9 and IL-27 cytokines were higher within the TME (*N* = 6) compared to the LNME (*N* = 6). Levels of soluble bFGF are higher within the tumour microenvironment compared with the LN microenvironment whereas, PIGF is higher within the lymph node microenvironment (*n* = 6) than the tumour microenvironment of OAC patients (*n* = 6). Tumour-draining lymph node (*n* = 6) and tumour tissue biopsies (*n* = 6) from OAC patients were cultured for 24h ex vivo to generate lymph node conditioned media (LNCM) and tumour conditioned media (TCM), respectively. The LNCM and TCM was screened for a panel of pro-angiogenic and vascular damage mediators using a 54-multiplex assay. LNCM = lymph node conditioned media, TCM = tumour. LN: lymph node and T: tumour.

Table S1. Analyte in matched tumour and lymph node.

Analyte	Tumour pg/ml (SD)	Lymph Node pg/ml (SD)	P Value n=6 per group Wilcoxon matched-pairs sign rank test
**Angiogenesis**			
VEGF-C	19.87 (12.5)	12.21 (8.576)	0.44
VEGF-D	17.87 (25.82)	23.92 (18.67)	0.68
CRP	6532 (10565)	7783 (6127)	1
ICAM-1	17082 (29191)	13791 (15118)	0.69
SAA	467.4 (351)	1797 (1041)	0.07
VCAM-1	21811 (39119)	18926 (24064)	0.69
**Cytokines, Chemokines and Growth Factors**			
IL-10	553.8 (503.9)	728.7 (557.2)	0.43
IL-12p70	10.14 (7.28)	15.52 (8.99)	0.21
IL-13	12.28 (4.91)	33.75 (37.33)	0.27
IL-1Beta	28.15 (35.64)	25.73 (52.2)	0.98
IL-2	4.044 (2.39)	4.76 (2.18)	0.69
IL-4	5.79 (4.27)	9.31 (4.58)	0.16
IL-6	1049 (892.7)	1513 (756.8)	0.22
IL-8	1420 (517.3)	1364 (635.2)	0.84
TNF-Alpha	11.02 (6.52)	10.06 (5.84)	0.98
GM-CSF	51.1 (85.44)	33.97 (66.85)	0.44
IL-12p40	48.51 (77.96)	38.57 (51.39)	0.84
IL-15	1.71 (1.91)	1.01 (1.22)	0.98
IL-16	919.6 (1372)	1690 (1241)	0.44
IL-17A	18.03 (25.08)	19.88 (19.24)	0.84
IL-1a	56.81 (101.2)	23.24 (42.84)	0.44
IL-5	1.29 (1.92)	1.13 (1.31)	0.56
IL-7	1.86 (1.09)	4.77 (6.92)	0.43
TNF Beta	1.12 (1.99)	1.31 (2.32)	0.31
IL-17A/F	8.45 (7.3)	4.44 (7.98)	0.69
IL-17B	8.08 (7.06)	2.232 (2.145)	0.13
IL-17C	15.94 (19.33)	4.24 (2.94)	0.16
IL-17D	46.29 (45.92)	48.95 (30.54)	0.97
IL-1RA	3268 (1789)	1623 (1851)	0.56
IL-3	28.69 (22.91)	10.91 (9.47)	0.31
TSLP	20.42 (19.53)	34.38 (35.83)	0.43
IL-21	0.32 (0.28)	1.09 (1.14)	0.06
IL-22	3.83 (6.4)	128 (334.7)	0.69
IL-23	1.26 (0.92)	4.06 (7.78)	0.84
IL-31	0.07 (0.05)	0.1 (0.08)	0.31
MIP-3a	280.2 (579)	211 (330.3)	0.69
